# SOAT1 methylation is associated with coronary heart disease

**DOI:** 10.1186/s12944-019-1138-9

**Published:** 2019-11-04

**Authors:** Jialin Abuzhalihan, Yong-Tao Wang, Yi-Tong Ma, Zhen-Yan Fu, Yi-Ning Yang, Xiang Ma, Xiao-Mei Li, Fen Liu, Bang-Dang Chen

**Affiliations:** 1 Department of Cardiology, First Affiliated Hospital of Xinjiang Medical University, Urumqi, 830054 People’s Republic of China; 2 Xinjiang Key Laboratory of Cardiovascular Disease Research, Urumqi, 830054 People’s Republic of China

**Keywords:** DNA methylation, Coronary heart disease, SOAT1, Cholesterol absorption

## Abstract

**Background:**

This study was designed to investigate whether differential DNA methylationin of cholesterol absorption candidate genes can function as a biomarker for patients with coronary heart disease (CHD).

**Methods:**

DNA methylation levels of the candidate genes FLOT1, FLOT2 and SOAT1 were measured in peripheral blood leukocytes (PBLs) from 99 patients diagnosed with CHD and 89 control subjects without CHD. A total of 110 CPG sites around promoter regions of them were examined.

**Results:**

Compared with groups without CHD, patients with CHD had lower methylation levels of SOAT1 (*P*<0.001). When each candidate genes were divided into different target segments, patients with CHD also had lower methylation levels of SOAT1 than patients without (*P* = 0.005). After adjustment of other confounders, methylation levels of SOAT1 were still associated with CHD (*P* = 0.001, OR = 0.290, 95% CI: 0.150–0.561).

**Conclusions:**

SOAT1 methylation may be associated with development of CHD. Patients with lower methylation levels in SOAT1 may have increased risks for CHD. Further studies on the specific mechanisms of this relationship are necessary.

## Background

Cholesterol is an important structural component of cell membranes, and a precursor to the synthesis of many bioactive molecules, including bile acids and salts, vitamin D, and steroid hormone [1]. In human body, the main source of cholesterol is from Intestinal absorption, and less than half of cholesterol is from in vivo synthesis. Within the cells, the cholesterol homeostasis is tightly regulated to ensure normal cellular processes [2, 3]. Previous studies have proved that mammals cannot survive without cholesterol, and the high concentration of blood cholesterol level leads to a series of diseases such as atherosclerosis, fatty liver, and coronary heart disease (CHD), which is a leading cause of death in developed countries [4–6]. Epidemiological studies have demonstrated that there are some risk factors, including genetic, environmental, behavioral, and clinical factors, contributing to development of CHD, but how these risk factors impact on the cellular level to cause coronary heart disease is not fully understood [7].

DNA methylation is a biological process by which a methyl group is added to a DNA nucleotide, without changing the underlying DNA sequence [8]. Methylated DNA causes the less effective combination of DNA binding proteins to the DNA’s main spiral groove. Therefore, it can regulate gene expression without changing DNA base sequence. The state of DNA methylation of CpG loci near gene promoters was found to be associated with transcriptional activity and gene expression [9, 10]. Recent studies on cardiovascular epigenetics showed that methylation of repetitive sequences, such as long-interspersed nucleotide repetitive elements-1 (LINE-1) and ALU have been associated with cardiovascular disease (CVD) [6]. Specifically, elevated methylation of ALU in leukocytes was associated with prevalence of CVD and obesity in Chinese individuals [11]. Patients with heart failure were found to have altered promoter methylation in genes relevant to myocyte apoptosis, fibrosis, and altered contractility [12].Comprehensive studies suggested that changes in DNA methylation states contribute to serious of disease such as dyslipidemia, atherosclerosis, hypertension, and inflammation [13]. The relationship between DNA methylation and coronary heart disease needs further investigation.

Growing evidence indicated that genetic factors may affect the cholesterol absorption in patients with cardiovascular diseases (CVD) [14]. Previous studies have suggested that many of genes such as FLOT1, FLOT2 and SOAT1, were closely associated with the cholesterol absorption [15–17]. We hypothesized that DNA methylation in some of these genes may reduce the absorption of cholesterol, and influence on the development of CHD. The aim of this study was to investigate whether DNA methylation has differences in patients with coronary heart disease from control group.

## Methods

### Study population

This study was approved by the Ethical Review Board of The First Affiliated Hospital of Xinjiang Medical University. Written informed consent was obtained from all enrolled patients. Patients with coronary heart disease were screened from The First Affiliated Hospital of Xinjiang Medical University between July 2012 and March 2016. Patients were included in the CHD group if they: (1) had previous coronary stent surgery histories; (2) aged 25 years or older; (3) Coronary angiography indicated at least one coronary artery was narrowed ≥75% or occluded. Patients were included in the control group if they: (1) had no previous coronary heart disease; (2) were not using Lipid-lowering drugs. Patients with malignant neoplasm, severe heart or liver, kidney dysfunction, autoimmune diseases were excluded. A total of 188 patients were finally enrolled in the present study.

### DNA isolation and genotyping

Venous blood samples were drawn in the morning after an overnight fasting for biochemical marker assaying and methylation analyzing. Genomic DNA was extracted from whole blood with commercially available kits (TIANGEN Biotech, Shanghai, China). DNA was quantified and then diluted to a working concentration of 20 ng/UL for genotyping.

CpG islands located in the proximal promoter of FLOT1, FLOT2 and SOAT1, were selected for measurement according to the following criteria: (1) 200 bp minimum length; (2) 50% or higher GC content; (3) 0.60 or higher ratio of observed/expected dinucleotides CpG. BiSulfite Amplicon Sequencing (BSAS) was used for quantitative methylation analysis [18]. Bisulfite conversion of 1 μg genomic DNA was performed with the EZ DNA Methylation™-GOLD Kit (ZYMO RESEARCH, CA, USA) according to the manufacturer’s protocol. Sodium bisulfite preferentially deaminates unmethylated cytosine residues to thymines, whereas methyl-cytosines remain unmodified. After PCR amplification (HotStar Taq polymerase kit, TAKARA, Tokyo, Japan) of target CpG regions and library construction, the products were sequenced on Illumina MiSeq Benchtop Sequencer (CA, USA). Primer sequences used for PCR were shown in Additional file 1: Table S1. All samples achieved a mean coverage of >600X. Each tested CpG site was named as its relative distance (in bp) to transcriptional start site (TSS). Methylation level at each CpG site was calculated as the percentage of the methylated cytosines over the total tested cytosines. The average methylation level was calculated using methylation levels of all measured CpG sites within the gene.

### Statistical analysis

The data were analyzed by IBM SPSS Statistics Version 17.0 (Armonk, NY: IBM Corp.). The measurement data are shown as the Means±SD, and the differences between CHD and control subjects were assessed using an independent-sample t-test. Differences in the enumeration data, such as the frequencies of smoking, drinking, hypertension and diabetes between CHD and control subjects were analyzed using the chi-square test. As the methylation levels of FLOT1, FLOT2 and SOAT1 did not meet the normality assumption, they were described as median (interquartile range) and compared with Mann–Whitney U test. These analyses were performed on both the average gene methylation data and the methylation data of the individual CpG loci. Additionally, logistic regression analyses with effect ratios (odds ratio [OR] and 95% CI) were used to assess the major risk factors of CHD. A two-tailed value of *p* < 0.05 was considered statistically significant.

## Results

Baseline characteristics and major risk factors for cardiovascular diseases were listed in Table 1. The average age of the 188 analyzed patients was 59.46 ± 10.61 years, and 127(67.6%) of them were male. There were 60(31.9%) patients with hypertension and 18(9.6%) with DM.

**Table 1:** Comparison of baseline characteristics between patients with and without CHD

Characteristics	All (*n* = 188)	CHD		*P* value
		With (*n* = 99)	Without (*n* = 89)	
Age, years	59.46 ± 10.61	59.23 ± 10.70	59.72 ± 10.60	0.754
Male, n (%)	127 (67.6%)	68 (68.7%)	59 (66.3%)	0.726
HTN, n (%)	60 (31.9%)	40 (40.4%)	20 (22.5%)	*0.008*
DM, n (%)	18 (9.6%)	14 (14.1%)	4 (4.5%)	0.025
Smoking, n (%)	72 (38.3%)	44 (44.4%)	28 (31.5%)	0.067
Drinking, n (%)	39 (20.7%)	24 (24.2%)	15 (16.9%)	0.212
TC, mmol/L	4.28 ± 1.00	4.52 ± 1.17	4.02 ± 0.69	*0.001*
TG, mmol/L	1.55 ± 0.71	1.65 ± 0.62	1.43 ± 0.78	*0.034*
HDL, mmol/L	1.14 ± 0.29	1.15 ± 0.24	1.12 ± 0.33	0.504
LDL, mmol/L	2.67 ± 0.41	2.74 ± 0.41	2.59 ± 0.39	*0.013*

Statistically significant values are in italics

Data are presented as number of patients (%) or mean standard±deviation

*CHD* Coronary artery disease, *HTN* Hypertension, *DM* Diabetes mellitus, *TC* Total cholesterol, *TG* Triglyceride, *HDL* High-density lipoprotein, *LDL* Low-density lipoprotein

Based on the above inclusion criteria, 99 patients were grouped as with and 89 without CHD. Compared with patients without CHD, those with CHD had higher prevalence of HTN (40.4% vs. 22.5%, *P* = 0.008) and T2DM (14.1% vs. 4.5%, *p* = 0.025). Patients with CHD had higher TC (4.52 vs. 4.02 mmol/L, *P* = 0.001), LDL (2.74 vs. 2.59 mmol/L, *P* = 0.013), TG (1.65 vs. 1.43 mmol/L, *P* = 0.034) than those without CHD (Table 1).

According to the results measured from target regions, there were 110 CpG sites (67 in FLOT1, 17 in FLOT2 and 26 in SOAT1) identified as methylated sites (detailed information of each site was shown in Additional file 1: Table S2).The distribution of methylation levels of the 142 CpGs were listed in Additional file 1: Table S3.

The methylation levels of each CpG sites compared between patients with and without CHD (Table 2). The methylated levels of 2 sites in FLOT1 and FLOT2, were higher in CHD group than that in control group respectively. The methylated levels of 7 sites in SOAT1 methylated levels were higher in control group than that in CHD group.

**Table 2:** Methylation levels (%) of candidate genes between patients with and without CHD

Gene	Position	CHD		*P* value
		With	Without	
FLOT1	45	0.55 (0–2.4)	0.41 (0–2.7)	*0.005*
	71	1.75 (0.2–3.2)	1.62 (0–6.7)	*0.049*
FLOT2	113	1.15 (0–3.4)	0.98 (0–3.3)	*0.041*
	155	1.93 (0–6.4)	1.73 (0–5.9)	*0.041*
SOAT1	61	18.81 (13.2–26.5)	19.67 (11.2–25.8)	*0.008*
	73	14.98 (10.4–20 .5)	15.94 (9.0–20.8)	*0.003*
	106	13.98 (12.0–16.2)	14.24 (11.4–16.0)	*0.027*
	165	27.67 (23.6–33.1)	28.46 (22.8–31.7)	*0.001*
	197	20.56 (15.6–26.9)	21.38 (15.2–25.5)	*0.003*
	205	16.03 (11.4–21.3)	16.47 (11.5–20.3)	*0.037*
	209	16.62 (13.0–20.8)	17.42 (12.5–21.0)	*0.001*

Statistically significant values are in italics

Differences in DNA methylation levels of each candidate genes between the two subgroups indicated that patients without CHD had higher methylation levels of SOAT1 than patients with CHD (*P*<0.001) (Table 3). When each candidate genes were divided into different target segments, patients without CHD also had higher methylation levels of SOAT1 than patients with (*P* = 0.005) (Table 4).

**Table 3:** Differences of methylation levels (%) between patients with and without CHD

Gene	CHD		*p* value
	With	Without	
FLOT1	1.64 (0.9–2.1)	1.61 (0.5–2.1)	0.611
FLOT2	1.78 (1.4–2.2)	1.76 (1.0–2.4)	0.592
SOAT1	6.09 (4.9–7.6)	6.79 (4.6–13.3)	*<0.001*

Statistically significant values are in italics

**Table 4:** Differences of methylation levels (%) between patients with and without CHD according to function segements

Target	CHD		*p* value
	With	Without	
FLOT1_1	1.73 (1.0–2.3)	1.72 (0–2.2)	0.908
FLOT1_2	0.78 (0.5–1.1)	0.84 (0–4.0)	0.463
FLOT1_3	1.83 (1.3–2.5)	1.88 (0–4.0)	0.241
FLOT1_4	3.35 (1.9–4.5)	3.29 (0–6.7)	0.717
FLOT2	1.78 (1.4–2.2)	1.76 (1.0–2.4)	0.592
SOAT1_1	0.70 (0.4–1.1)	0.70 (0–1.3)	0.569
SOAT1_2	14.71 (11.7–18.7)	15.21 (10.5–18.2)	*0.005*

Statistically significant values are in italics

Further, we assessed the association between SOAT1 methylation levels and CHD. After adjustment of male, age, smoking, drinking, hypertension, diabetes, TG, TC, HDL-C and LDL-C, SOAT1 methylation levels were still associated with CHD (*P* = 0.001, OR = 0.290, 95% CI: 0.150–0.561) (Table 5).

**Table 5:** Association of SOAT1 methylation levels and CHD after adjustment of other confounders

	OR	95%CI of OR	P value
Male	1.250	0.534–2.930	0.607
Age	1.007	0.973–1.042	0.690
Smoking	1.868	0.820–4.529	0.137
Drinking	0.833	0.317–2.187	0.711
Hypertension	1.778	0.830–3.809	0.139
Diabetes	3.442	0.827–14.317	0.089
TG	1.592	0.927–2.734	0.092
TC	1.578	1.058–2.354	*0.025*
HDL-C	2.010	0.570–7.087	0.278
LDL-C	1.811	0.755–4.345	0.184
SOAT1 methylation levels	0.290	0.150–0.561	*0.001*

Statistically significant values are in italics

## Discussion

In this study we analyzed DNA methylation levels of a subset of candidate genes in patients diagnosed with CHD and control subjects without CHD. All of the genes analyzed in this study were previously reported to have a potential importance in the absorption of the cholesterol [15–17, 19–21]. Our study observed that methylation levels of SOAT1 were relatively lower in patients with CHD than control group. These results verified our hypothesis that DNA methylation in SOAT1 plays vital role in the development of CHD.

Sterol O-acyltransferase (SOAT) is a key enzyme converting endoplasmic reticulum cholesterol to cholesterol esters, which are stored in the form of lipid droplet [22]. There are two SOAT isoforms in mammals, including SOAT1 and SOAT2, with different tissue expression patterns. SOAT1 is ubiquitously expressed in tissues and cells, while SOAT2 is predominantly expressed in the liver and intestines [23]. Reports indicated that SOAT1 is constitutively expressed and likely functions to maintain the intracellular balance of free and esterified cholesterol, whereas SOAT2 is associated with intestinal cholesterol absorption and hepatocyte-specific functions such as lipoprotein particle assembly and the production of bile, and SOAT1 is responsible for the formation of foam cell in macrophages [24, 25]. Previous studies also reported that SOAT1 was involved in the formation of atherosclerotic plaques, and was involved in regulating inflammation.

Some researchers have observed that hypercholesterolemia can cause multiple physiologic outcomes, such as coronary artery disease, diabetes and obesity [26, 27]. SOAT1 is an important protein for regulating cholesterol absorption and it plays a pivotal role in the development of atherosclerosis. Recent studies have shown that the deletion of the SOAT1 gene in macrophages results in an increase in atherosclerotic lesion area in the aortas of hypercholesterolemia mice [28, 29]. In 1996, the production of genetically engineered mice lacking functional SOAT1 was first reported by Farese et al. [30]. SOAT1 gene disruption resulted in decreased cholesterol esterification in fibroblasts and adrenals. Studies of global DNA methylation in CVD patients report both increased and decreased DNA methylation. In addition, DNA isolated from atherosclerotic tissue has been observed to be hypomethylated globally, however, DNA hypermethylation was observed in the promoter regions of genes associated with atherosclerosis [7, 31, 32]. Therefore, we assume that SOAT1 gene and coronary artery disease might be associated. However, few researches have been conducted regarding the relationship between SOAT1 gene and cardiovascular diseases. Human and mouse studies have observed DNA hypermethylation of cytosines in the context of CpGs as an accompanying feature of atherosclerosis [33]. Methylation in CpG islands near to promoter regions may promote gene expression. It was indicated in our study that hypermethylation of SOAT1 may promote gene expression and results in atherosclerosis.

Our study showed that these candidate genes have different levels of methylation. The methylation levels of each CpG site of them differ one from another. SOAT1 gene has more CpG methylation sites than others have. This may suggest that the more the number of methylation sites, the greater the contribution to gene expression regulation. When comparing methylation sites at the genetic level, SOAT1 gene had a significantly higher methylation levels than other genes had, and it was proven again that high methylation levels of SOAT1 significantly differentiate from others, when analyzing each of genes divided into different functional segments. Therefore, our study implied that DNA methylation of SOAT1 may play a potential role in cholesterol absorption and development of CHD. Further study is still indeed to enrich our outcomes.

There are several limitations in the present study. First, the source of CHD patients was limited to the First Affiliated Hospital of Xinjiang Medical University, and these subjects may possess some risk factors of cardiovascular disease, which may generate selection bias. Second, the relatively small sample size of this study may have contributed to weak statistical significance. Third, there is a lack of individual genetic background information for the subjects enrolled. Finally, the present study lacked functional validation. However, assessments of the functional outcomes and correlations with hormonal levels are currently ongoing. Nevertheless, a prospective cohort study with a reasonably long study duration is required to obtain evidence with higher quality and reliability.

## Conclusions

In summary, SOAT1 methylation may be associated with development of CHD. Patients with lower methylation levels in SOAT1 may have increased risks for CHD. Our findings may provide some new implications for prevention and treatment of atherosclerotic diseases. Further studies on the specific mechanisms of this relationship are necessary.

## Supplementary information

**Additional file 1: Table S1:** Primer sequences used for PCR. **Table S2:** Methylated CpG sites identified in this study. **Table S3:** Distribution of methylation levels (%) of 142 CpGs sites in candidate genes.

## Data Availability

The data will not be shared, since part of the data is being reused by another study.

## Abbreviations

CHD: Coronary heart disease
CVD: Cardiovascular disease
DM: Diabetes mellitus
HDL: High-density lipoprotein
HTN: Hypertension
LDL: Low-density lipoprotein
PBL: Peripheral blood leukocytes
TC: Total cholesterol
TG: Triglyceride
TSS: Transcriptional start site
